# Human Population Differentiation Is Strongly Correlated with Local Recombination Rate

**DOI:** 10.1371/journal.pgen.1000886

**Published:** 2010-03-26

**Authors:** Alon Keinan, David Reich

**Affiliations:** 1 Department of Genetics, Harvard Medical School, Boston, Massachusetts, United States of America; 2 Broad Institute of Harvard and Massachusetts Institute of Technology, Cambridge, Massachusetts, United States of America; 3 Department of Biological Statistics and Computational Biology, Cornell University, Ithaca, New York, United States of America; University of California Davis, United States of America

## Abstract

Allele frequency differences across populations can provide valuable information both for studying population structure and for identifying loci that have been targets of natural selection. Here, we examine the relationship between recombination rate and population differentiation in humans by analyzing two uniformly-ascertained, whole-genome data sets. We find that population differentiation as assessed by inter-continental *F*_ST_ shows negative correlation with recombination rate, with *F*_ST_ reduced by 10% in the tenth of the genome with the highest recombination rate compared with the tenth of the genome with the lowest recombination rate (P≪10^−12^). This pattern cannot be explained by the mutagenic properties of recombination and instead must reflect the impact of selection in the last 100,000 years since human continental populations split. The correlation between recombination rate and *F*_ST_ has a qualitatively different relationship for *F*_ST_ between African and non-African populations and for *F*_ST_ between European and East Asian populations, suggesting varying levels or types of selection in different epochs of human history.

## Author Summary

A common assumption when analyzing patterns of human genetic variation is that most of the genome can be treated as “nearly neutral,” in the sense that the effects of natural selection on allele frequencies are very small compared with the influence of population demographic history. To test the validity of this assumption, we analyzed data from more than a million human polymorphisms and summarized allele frequency differences across populations. We find that, compared with the genome-wide average, allele frequency differences are 7% reduced on average in the tenth of the genome with the highest recombination rate and are 3% increased in the tenth with the lowest rate. Such a correlation cannot be explained by demography. Instead, the pattern reflects the fact that forces of natural selection have had a profound impact on patterns of variation throughout the genome in the last 100,000 years.

## Introduction

Single Nucleotide Polymorphism (SNP) allele frequency differentiation between human populations, usually measured by Wright's *F*_ST_ [1],[2], has been extensively studied both for characterizing population structure and demographic history [3]–[10], and for detecting loci that have experienced the effects of natural selection [8], [11]–[15]. In a recent study, Barreiro et al. found that global, inter-continental human population differentiation has been shaped in an important way by natural selection, with negative selection reducing population differentiation and mostly affecting coding regions, and positive selection increasing population differentiation and affecting primarily SNPs that are nonsynonymous or in the 5′ untranslated regions of genes [16]. In the current study, we used the variability of recombination rate across the genome as a tool to learn about how substantially the forces of natural selection have shaped patterns of inter-continental allele frequency differentiation across populations. Nucleotides that are in regions of the genome with low recombination rates are expected to be more affected by natural selection (due to hitchhiking and background selection from sites in the vicinity) than nucleotides in regions of the genome with high recombination rates. By measuring the extent to which regions of high and low recombination rate differ in their *F*_ST_, it may be possible to learn about how much of the overall differentiation in allele frequencies across populations can be accounted for by natural selection.

Many studies have documented a positive correlation between *nucleotide diversity* and recombination rate in many species, notably in *Drosophila* [17]–[19], humans [10], [20]–[25], and *Arabidopsis lyrata* [26]. In *Drosophila*, humans, and maize, a positive correlation was also observed between interspecific sequence divergence and recombination rate [17], [22], [27]–[29]. The observed correlation between nucleotide diversity or divergence and recombination rate has often been ascribed to natural selection [18],[20],[21],[23],[24],[29],[30] since directional selection reduces diversity and its effect on linked neutral sites extends further in regions of lower recombination rate. Thus, both hitchhiking (associated with positive selection) [31] and background selection (the “hitchhiking” associated with negative selection) [32], are expected to give rise to a positive correlation between diversity and recombination rate [32]–[35]. A recent analysis in humans modeled the slightly different effects of hitchhiking and background selection on the shape of the correlation between nucleotide diversity and recombination rate and concluded that positive selection better explains the data [20], while another recent study concluded that either hitchhiking or background selection explain their results [22].

A substantial body of research has explored an alternative, mechanistic explanation for the observed positive correlation between nucleotide diversity and recombination, which is the mutagenic effect of recombination [10],[17],[21],[27],[36],[37],[38]. One way to partially disentangle the mutagenic effect of recombination from the effect of selection is to normalize nucleotide diversity by interspecific divergence such as human-chimpanzee divergence, while making the assumption that large-scale recombination rates and mutation rates have been unchanged since species divergence [10],[20],[22]. The two aforementioned recent genome-wide studies reported that a significant correlation of human nucleotide diversity with recombination rate is preserved following normalization by human-chimpanzee divergence [20],[22]. Another recent study that focused on 40 regions with medium to high recombination rate reported that a significant correlation of human diversity with recombination rate vanished following such normalization [10]. A secondary concern with the approach of normalizing by interspecific divergence is that it does not take into account the effect that hitchhiking and background selection may have had in the ancestral population of humans and chimpanzees [39]. This effect, which can contribute to the observed correlation between interspecific divergence and recombination rate, is expected to weaken the observed normalized correlation of human nucleotide diversity with recombination rate.

In this study, we examine the relationship between recombination rate and population differentiation in allele frequencies of ascertained polymorphisms. For this type of analysis, the mutagenic effect of recombination is not a confounder, and any observed correlation is expected to be the result of selection in human history since the split of the analyzed populations. Different types of natural selection are expected to affect patterns of allele frequency differentiation: Positive selection is predicted to produce a negative correlation between *F*_ST_ and recombination rate if adaptation is local and selective sweeps drive alleles to high frequency in some but not all of the populations between which *F*_ST_ is measured. Selective sweeps are expected to extend less far in regions of higher recombination rate, and thus allele frequency differentiation is expected to be higher on average in regions of low recombination rate [16],[30],[40],[41],[42],[43]. Negative (purifying) selection can also produce a negative correlation between *F*_ST_ and recombination rate. While negative selection is expected to decrease population differentiation at the site under selection itself [16],[40], background selection decreases the effective population size at sites linked to negatively selected alleles, thereby increasing their allele frequency differentiation between populations as captured by relative measures such as *F*_ST_ [41], [43]–[45]. As the effect of background selection extends further in regions of lower recombination rate, higher population differentiation on average is predicted in such regions. There is also another important difference between our approach of examining the relationship between population differentiation and recombination rate, to previous approaches of comparing nucleotide diversity and recombination rate. Population differentiation only reflects events that occurred since two populations split, and hence allows us to study the effects of natural selection over a circumscribed time period (as opposed to averaging over the hundreds of thousands of years of a population's history).

To empirically examine the correlation of recombination rate and allele frequency differentiation in human populations, we needed to examine data sets that were ascertained in a uniform way across the genome, in a manner that is independent of local recombination rate. We focused on two data sets: 1,110,338 Perlegen “class A” SNPs [46], as well as 248,886 uniformly-ascertained SNPs that we previously reported [4] and that are mostly a subset of the International Haplotype Map (HapMap) Phase II [9]. These two data sets allowed us to interrogate population differentiation between North Europeans, East Asians, African Americans, and West Africans. The aims of this paper are to probe the correlation between global population differentiation and recombination rate, and to test whether it is attributable to natural selection. Once we establish that a strong correlation exists and that it likely reflects a history of natural selection, we explore how the impact of selection has varied in different epochs of human history by characterizing the differentiation between different pairs of populations that separated at different times.

## Results

### Population differentiation is inversely correlated with recombination rate

Our primary set of analyses were carried out on 1,110,338 Perlegen “class A” autosomal SNPs [46]. We used this set of SNPs since it is the largest data set that we are aware of for which the ascertainment of SNPs is uniform across the genome. Perlegen “class A” SNPs were discovered by array-based resequencing of 24 human samples of diverse ancestry [46],[47]. While this ascertainment scheme is expected to introduce a complicated ascertainment bias in terms of allele frequency correlations across populations—since different SNPs were ascertained in different numbers of samples from different ancestries—this bias is expected to be the same at every point on the autosomes [46]. Due to the uniform nature of SNP ascertainment independent of recombination rate, any correlations that are observed between allele frequency patterns at these SNPs and recombination rate are not expected to be due to locus specific differences in SNP ascertainment. For each SNP, we estimated recombination rate in a 3 Mb stretch centered on the SNP based on the deCODE genetic map [48] (Methods), which results in a median across all SNPs of 1.23 cM/Mb (mean of 1.39 cM/Mb).

**Figure 1:**
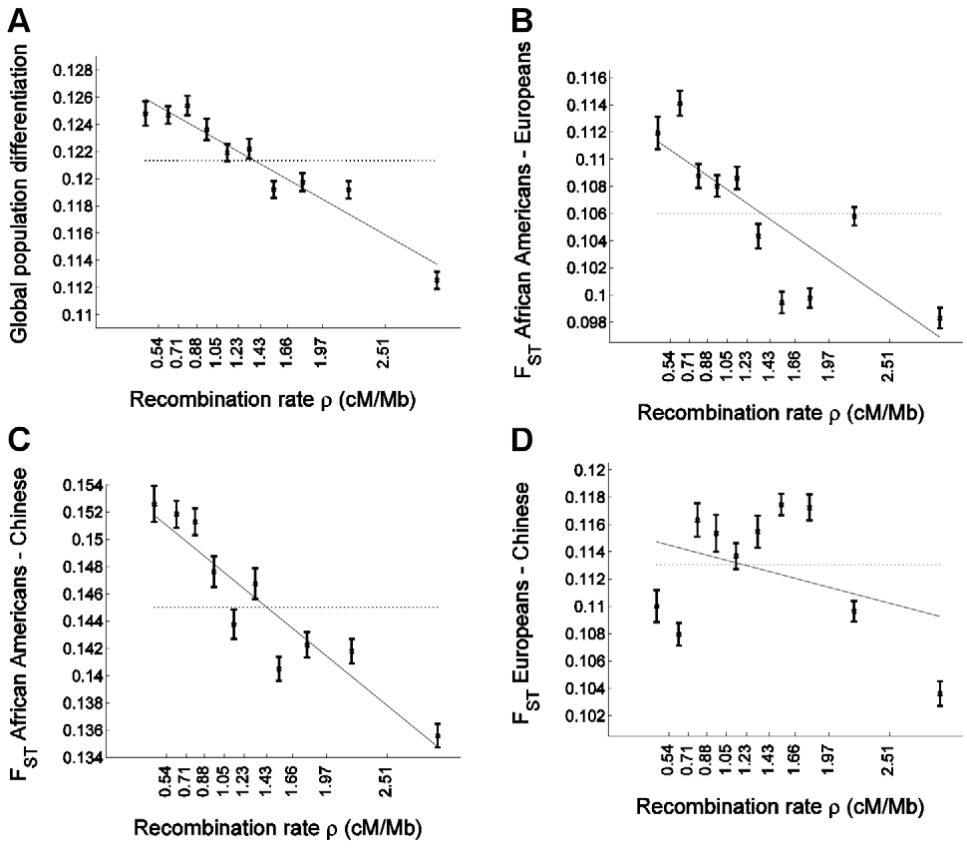
Population differentiation in allele frequencies is inversely correlated with recombination rate. We placed 1,110,338 SNPs into 10 bins according to the recombination rate in a 3 Mb window centered on each SNP. The x-axis of all panels indicates the recombination rate, with the values indicated on the ticks corresponding to the edges between 10 bins. For each bin, at an x-axis position corresponding to the median recombination rate across the SNPs at that bin, the figure presents (A) global population differentiation between African Americans, Europeans, and Chinese; (B) *F*_ST_ between African Americans and Europeans; (C) *F*_ST_ between African Americans and Chinese; and (D) *F*_ST_ between Europeans and Chinese. Error bars indicate ±1 standard error, which is estimated based on 1,000 moving block bootstraps over the SNPs in the bin. Linear regression of *F*_ST_ estimates as a function of median recombination rate in each bin is also presented (solid line) and corresponds to (A) 0.1280–0.0048ρ (B) 0.1138–0.0057ρ (C) 0.1546–0.0067ρ and (D) 0.1156–0.0022ρ. The corresponding correlation coefficient estimates between *F*_ST_ and median recombination rate are (A) r = −0.962 (P = 8.9×10^−6^), (B) −0.815 (P = 0.0041), (C) −0.931 (P = 0.0001), and (D) −0.361 (P = 0.306). For comparison, population differentiation based on all SNPs in all bins combined is also presented (horizontal dotted line). The y-axis range is different between the four panels but spans 0.02 units in all. Figure S1 repeats Figure 1A for sets of SNPs of different minor allele frequency categories.

To examine whether and how population differentiation depends on recombination rate, we assigned SNPs to equally-sized bins according to the recombination rate around them. For each bin, we estimated global population differentiation for the SNPs in that bin as the *F*_ST_ between the three population samples, which consisted of 24 individuals of European ancestry, 24 individuals of Han Chinese ancestry, and 23 African Americans [46]. Population differentiation shows a strong dependence on recombination rate, ranging from 0.1248±0.0009 for the SNPs in the bin of lowest recombination rate to 0.1125±0.0006 for the SNPs in the bin of highest recombination rate, and 0.1213 for all SNPs combined (Figure 1A). More generally, we find a striking correlation between population differentiation of SNPs in a bin and the recombination rate in that bin, with a correlation coefficient between the two of r = −0.96 (P = 8.9×10^−6^). A linear regression of population differentiation as a function of recombination rate provides a reasonably good fit to the data, and predicts an average decrease of 0.0048 (4%) in *F*_ST_ for every 1 cM/Mb increase in recombination rate (Figure 1A). As binning the data by recombination rate averages out some of the variability across individual SNPs, we repeated the analysis without binning and observed a very significant correlation also between *F*_ST_ of single SNPs and recombination rate around them (r = −0.015; P≪10^−12^).

### Statistical framework for relating population differentiation and recombination rate

To characterize in more detail the relationship between population differentiation and recombination rate, we designed a more elaborate statistical framework that overcame three limitations of the correlation and regression analysis described above: (i) The previous analysis did not incorporate uncertainty in *F*_ST_ estimation that is due to a limited number of SNPs in each bin. (We did estimate standard errors in each bin via bootstrapping as presented in Figure 1, but did not incorporate these errors into the inference of the relationship between *F*_ST_ and recombination rate.) (ii) Because the regression did not incorporate this uncertainty, we could not apply the analysis to many recombination rate bins since as the number of SNPs per bin decreased the noise obscuring the signal increased. (iii) The analysis did not account for correlation between SNPs due to linkage disequilibrium (LD), which is important especially since LD is itself correlated to recombination rate.

To overcome these limitations, we developed a bootstrapping framework for estimating several statistics that capture the relationship between recombination rate and population differentiation. The framework generates many data sets of the same size as the original using a Moving Block Bootstrap (MBB) [49],[50],[51]. The MBB works by resampling contiguous runs of SNPs from the data (with replacement) to appropriately take into account the effect of correlation between SNPs due to LD [4]. Within each bootstrap, our statistical framework places the randomly selected set of SNPs into their associated recombination rate bins, estimates population differentiation for the SNPs, and measures correlation and regression statistics between differentiation and recombination rate along the same lines as the analysis described above. We estimated statistics by averaging across many bootstraps and estimated standard errors as the standard deviation of the estimates across bootstraps. Our estimates thus account for uncertainty in population differentiation estimation and also account for dependence among neighboring SNPs. They thus provide standard errors that we can use for hypothesis testing.

After applying the bootstrapping framework to the data set, the correlation between *F*_ST_ and recombination rate remains extremely significant: r = −0.89±0.06 (P≪10^−12^). Linear regression of *F*_ST_ as a function of recombination rate results in a best fitting relationship of 0.1280–0.0049ρ, which is very similar to the naïve analysis; but, importantly, the bootstrapping framework allows us to perform hypothesis testing based on the standard error estimates across bootstraps (Table 1). In particular, the recombination rate regression coefficient of −0.0049±0.0007 is significantly different from zero (P = 2.6×10^−12^) as is a t-statistic for the significance of the linear regression coefficient: −6.09±2.18 (P = 0.0051; Table 1). This further supports the conclusion that recombination rate is a significant explanatory variable of population differentiation. Increasing the number of recombination rate bins from 10 to 40 yielded similar results (Table 1), except that the greater number of bins allows for a more consistent application of the regression analysis, resulting in a more significant departure of the t-statistic from zero: −5.45±0.99 (P = 3.5×10^−8^). We use 10 recombination rate bins in all following analyses.

**Table 1:** Bootstrapped correlation and regression coefficient estimates of *F*_ST_ as a function of recombination rate in Perlegen data.

	Global *F*_ST_				Pairwise *F*_ST_		
	All SNPs	All SNPs (40 bins)	Coding SNPs	Non-coding SNPs	AA vs. Europeans	AA vs. Chinese	Europeans vs. Chinese
Number of SNPs	1,110,338	1,110,338	21,391	1,088,947	1,110,338	1,110,338	1,110,338
*b_0_* ± stderr (p-value)	0.1280±0.0012 (≪10^−12^)	0.1277±0.0011 (≪10^−12^)	0.1381±0.0038 (≪10^−12^)	0.1279±0.0012 (≪10^−12^)	0.1137±0.0014 (≪10^−12^)	0.1547±0.0015 (≪10^−12^)	0.1156±0.0018 (≪10^−12^)
*b_1_* ± stderr (p-value)	−0.0049±0.0007 (2.6×10^−12^)	−0.0046±0.0007 (5.0×10^−11^)	−0.0081±0.0022 (2.3×10^−4^)	−0.0048±0.0007 (7.0×10^−12^)	−0.0057±0.0008 (1.0×10^−12^)	−0.0067±0.0010 (2.1×10^−11^)	−0.0021±0.0010 (0.036)
*r* ± stderr (p-value)	−0.8862±0.0567 (≪10^−12^)	−0.6476±0.0678 (≪10^−12^)	−0.6801±0.1286 (1.2×10^−7^)	−0.8823±0.0561 (≪10^−12^)	−0.7697±0.0589 (≪10^−12^)	−0.8448±0.0635 (≪10^−12^)	−0.3244±0.1544 (0.036)
*t* ± stderr (p-value)	−6.0926±2.1758 (0.0051)	−5.4512±0.9889 (3.5×10^−8^)	−2.8775±1.0702 (0.0072)	−5.8950±1.9773 (0.0029)	−3.5253±0.6955 (4.0×10^−7^)	−4.7959±1.2800 (1.8×10^−4^)	−1.0147±0.5389 (0.0597)
*b_1_*/*b_0_* ± stderr	−0.0379±0.0052	−0.0358±0.0049	−0.0585±0.0147	−0.0376±0.0053	−0.0499±0.0064	−0.0430±0.0059	−0.0185±0.0088

To characterize the correlation between *F*_ST_ and recombination rate, we applied the MBB-based bootstrapping framework (Methods) in several scenarios. These scenarios include considering global *F*_ST_ between all three populations genotyped in the Perlegen data set, as well as considering pairwise *F*_ST_. For the global *F*_ST_ (first four columns), we performed an analysis on all SNPs based on 10 and 40 recombination rate bins (all other analyses are with 10 bins), and performed separate analyses of coding SNPs and non-coding SNPs. For pairwise *F*_ST_ (last three columns), we repeated the analysis for *F*_ST_ between each pair of populations (AA in column heading stands for African Americans). For each analysis, the table reports five summaries of the correlation between *F*_ST_ and recombination rate: The regression coefficients of *F*_ST_ = *b_0_*+*b_1_ρ*, where *F*_ST_ is estimated across all SNPs in a bin and *ρ* is the median recombination rate in that bin, the correlation coefficient *r* across bins between *F*_ST_ and *ρ*, a t-statistic *t* for the significance of the linear coefficient of the above regression, and a normalized regression coefficient *b_1_*/*b_0_*. For each of these statistics, the average and standard deviation (stderr) across 1,000 bootstraps is reported, as well as a p-value based on a two-sided z-test for the mean being different from zero (except for the last statistic). When comparing between different scenarios (columns), we note that comparing *b_1_* alone does not account for *F*_ST_ being generally higher in one scenario than the other, and thus we compare *b_1_*/*b_0_*, which captures the predicted relative change in *F*_ST_ as a function of recombination rate. We also note that *r* and *t* cannot be compared between some of the different scenarios since they depend on the number of SNPs analyzed.

### Stronger impact of recombination rate on population differentiation in genes

We cannot envision any demographic or mechanistic explanation that would produce a correlation between recombination rate and allele frequency differentiation as observed and we hypothesize that our observations reflect a history of natural selection. Natural selection is usually expected to increase population differentiation at linked neutral sites [16],[30],[40],[41],[42],[43],[44],[45], an effect that is expected to extend over longer physical distances in regions of lower recombination rate. A prediction of an explanation based on natural selection is that the effect would be more marked in regions that are more likely to be influenced by selection, such as genes. To test this prediction, we identified a subset of 21,391 SNPs in the data that are in coding exons (cSNPs) and first considered the differentiation of this subset of SNPs without regard to recombination rate. Consistent with the results of Barreiro et al. [16], we found that the set of cSNPs exhibits a significantly higher (P = 9.0×10^−4^) population differentiation: 0.1265±0.0016 compared with 0.1212±0.0006 for non-coding SNPs (ncSNPs).

**Figure 2:**
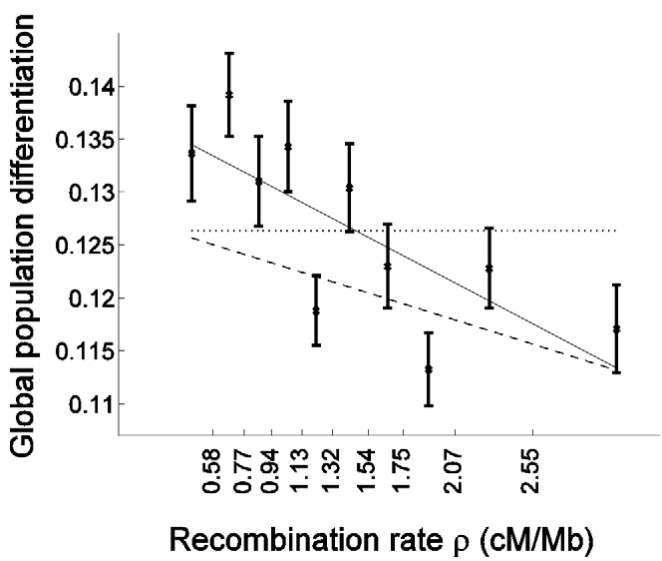
Population differentiation is more strongly correlated with recombination rate in genes than outside of genes. Global population differentiation between African Americans, Europeans, and Chinese is presented for coding SNPs (cSNPs). Except for focusing on the 21,391 SNPs in coding exons, the figure is identical to Figure 1A. In addition to the linear regression of *F*_ST_ estimates as a function of the median recombination rate in each bin (solid line; 0.1381–0.0081ρ), the linear regression for the rest of the data set (non-coding SNPs) is provided (dashed line; 0.1278–0.0048ρ), which is very similar to the regression based on the entire data set (Figure 1A). The correlation coefficient between *F*_ST_ of cSNPs and median recombination rate is −0.752 (P = 0.012).

The novel signal of a negative correlation to recombination rate that we observed is more pronounced in genes: The slope of the regression of *F*_ST_ as a function of recombination rate is steeper for cSNPs than for ncSNPs (Figure 2). Interestingly, the regression predicts that for very high recombination rate, where the hitchhiking/background selection effect is expected to be weak, population differentiation is similar between cSNPs and ncSNPs (Figure 2). This suggests that selection may be the driving force behind both the higher population differentiation generally observed in genes and the correlation of population differentiation and recombination rate.

To test formally whether selection has an impact on the correlation with recombination rate above and beyond the general effect of increased population differentiation in genes, while controlling for the characteristically different recombination rate in genes, we repeated the bootstrapping framework analysis on cSNPs and compared it with analysis of ncSNPs (Table 1). As expected from the higher differentiation in genes, the constant coefficient of the linear regression is larger for cSNPs (Table 1; P = 0.005). Furthermore, as predicted if selection explains the correlation with recombination rate, the regression's slope is also steeper for cSNPs, −0.0081±0.0022 vs. −0.0048±0.0007, though due to the limited number of cSNPs this result is only borderline significant (P = 0.076; Table 1). To account for the possibility that the regression slope is steeper merely due to the differentiation in genes being generally higher, we compared the slope normalized by the regression constant coefficient, which captures the percentage change in population differentiation for each 1 cM/Mb (Table 1). This normalized slope is also steeper in genes (−0.0585±0.0147 vs. −0.0376±0.0053), though the result is only suggestive (P = 0.09).

### Characteristically different correlation for different population pairs

We next considered the effect of recombination rate on population differentiation between each pair of populations separately. A negative correlation is observed between all pairs of populations, but the pattern is qualitatively different across population pairs (Figure 1B–1D and Table 1). The regression predicts a larger effect size, as assessed by the percentage change in *F*_ST_ per cM/Mb (to account for the varying levels of population differentiation between different populations), for *F*_ST_ between African Americans and Europeans and for *F*_ST_ between African Americans and Chinese, than for *F*_ST_ between Europeans and Chinese (P = 0.004 and P = 0.021; Table 1). We observed no significant difference in effect size for *F*_ST_ between African Americans and Europeans and for *F*_ST_ between African Americans and Chinese (P = 0.43; Table 1). When we considered single SNPs, without binning the data by recombination rate, we observed a very significant correlation with recombination rate for African American–European *F*_ST_ (r = −0.0183; P≪10^−12^), as well as for African American–Chinese *F*_ST_ (r = −0.0147; P≪10^−12^), but no correlation with recombination rate for European–Chinese *F*_ST_ (r = 0.0002; P = 0.81).

The weaker correlation for the *F*_ST_ between European and Chinese populations is driven by a dip in differentiation at very low recombination rate loci (Figure 1D), which is not at all what is seen in the comparison of African and non-African populations (Figure 1B and 1C). This curve shows a qualitatively non-monotonic pattern, which motivated us to perform a quadratic regression fitted within the bootstrapping framework. The regression is concave and includes very significant linear (P = 3.0×10^−4^) as well as quadratic (P = 1.8×10^−5^) terms. Conversely, quadratic regression gives a non-significant quadratic term for *F*_ST_ between African Americans and each of the other two populations and if anything is slightly convex. As expected, for single SNP analysis (without binning by recombination rate), linear regression is very significant for *F*_ST_ between African Americans and either non-African population (P≪10^−12^). For *F*_ST_ between Chinese and Europeans, however, linear regression is not significant (P = 0.81), while a quadratic regression is very significant (P≪10^−12^).

These results suggest a qualitatively different effect of recombination rate on allele frequency differentiation for different pairs of human populations and in different epochs of human history. In particular, most of the signal we observed of a correlation between recombination rate and *F*_ST_ of all three populations (Figure 1A) is attributable to selection that occurred either on the non-African lineage before the split of Europeans and Chinese or on the African lineage. While African American allele frequencies are a mixture of African and European allele frequencies, which are hence averaged in this analysis, a non-monotonic pattern such as observed between Europeans and Chinese is not observed in comparisons of African Americans and non-Africans.

### Replication with uniformly ascertained subsets of HapMap

Considering the complexity of the ascertainment scheme [46], it is plausible that some features of the ascertainment of Perlegen “class A” SNPs would be correlated with recombination rate. In particular, though an effort has been made to mask repeat intervals [46], it is still possible that repeat content and GC content affect sequencing depth in the ascertainment panel [52]. If the same genomic features are also correlated with recombination rate, this could affect the correlation between *F*_ST_ and recombination rate. However, GC and repeat content are expected if anything to produce a correlation in the opposite direction to the one we observed, as these features are associated with lower resequencing depth, which would be expected to bias SNPs toward having a higher minor allele frequency. High minor allele frequency SNPs are empirically observed to be more differentiated than lower frequency SNPs (Figure S1), which would be expected to generate a positive correlation between recombination rate and *F*_ST_ since these features are associated with higher recombination rate.

**Figure 3:**
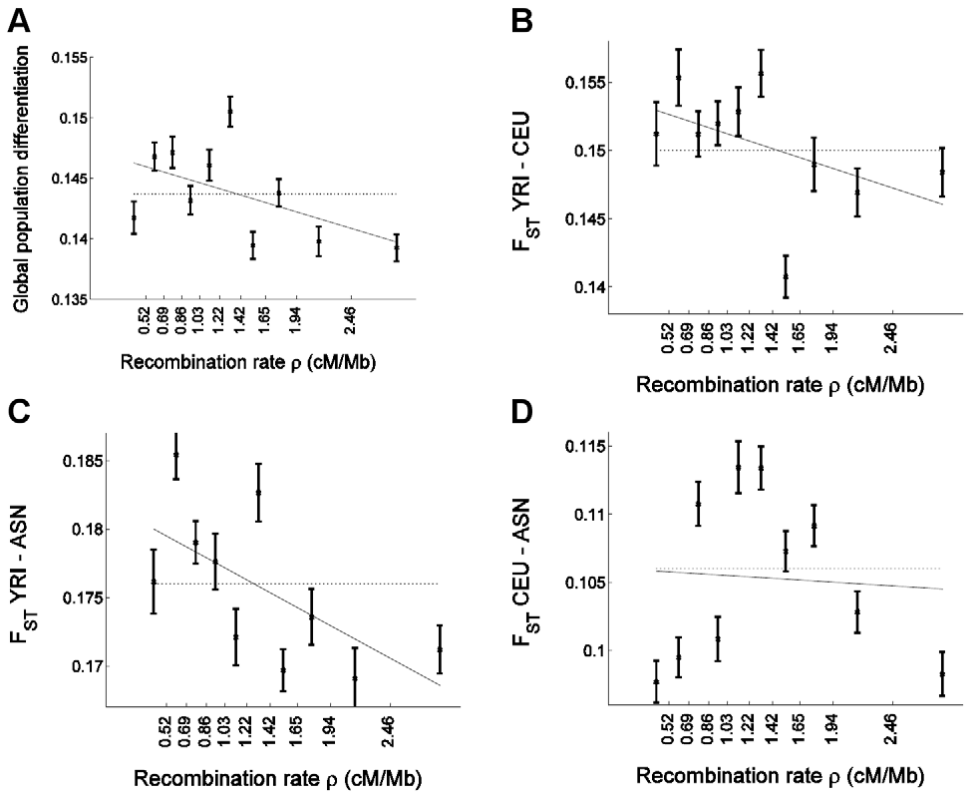
Confirmation of the correlation of allele frequency differentiation and recombination rate in uniformly-ascertained subsets of HapMap. Similar to Figure 1, we placed 248,886 uniformly-ascertained HapMap SNPs into 10 bins according to the recombination rate and estimated for each bin (A) global population differentiation between YRI, CEU, and ASN (ASN denotes the combined CHB and JPT samples); (B) *F*_ST_ between YRI and CEU; (C) *F*_ST_ between YRI and ASN; and (D) *F*_ST_ between CEU and ASN. Linear regression as a function of the median recombination rate (solid line) is (A) 0.1473–0.0026ρ (B) 0.1541–0.0028ρ (C) 0.1819–0.0046ρ, and (D) 0.1060–0.0005ρ. The corresponding correlation coefficient estimate between *F*_ST_ and median recombination rate is (A) r = −0.526 (P = 0.118), (B) −0.482 (P = 0.158), (C) −0.634 (P = 0.049), and (D) −0.066 (P = 0.857).

To replicate our results in an independent data set with less complex ascertainment, we applied similar analyses on a data set of uniformly-ascertained SNPs that we previously reported, where ascertainment was carried out in two chromosomes of known ancestry in a way that is independent of the effect of genomic features on coverage and where the discovery in two chromosomes cannot result in a frequency bias associated with recombination rate [4],[5]. This data set is mostly composed of subsets of SNPs from HapMap II and for which genotype information is available in 60 unrelated West Africans (YRI), 60 unrelated European Americans (CEU), and 90 unrelated East Asians (ASN, denoting the combined CHB and JPT samples). Another advantage of this data set, in addition to the simplified ascertainment process, is that it includes a West African population, as opposed to the African American population of the Perlegen data set, for which analysis has been complicated by the admixture of European ancestry. We estimated recombination rate around 248,886 such uniformly-ascertained autosomal HapMap SNPs, which we then used to repeat all analyses that we had applied in the previous sections to the Perlegen SNPs (Figure 3; Table S1).

Results from the analysis with uniformly-ascertained subsets of HapMap replicated the previous results based on the Perlegen data. In particular, we replicated the strong negative correlation between global population differentiation and recombination rate (Figure 3A), and obtained a significant negative correlation coefficient (P = 0.001) and recombination rate regression coefficient (P = 0.004) based on the bootstrapping framework (Table S1). Furthermore, similar to the pattern in the Perlegen data set, we observed significant linear correlations with recombination rate for pairwise *F*_ST_ between YRI and CEU (P = 0.008 for correlation and P = 0.024 for regression coefficient) and for *F*_ST_ between YRI and ASN (P = 1.0×10^−5^ and P = 3.0×10^−4^), but not for *F*_ST_ between CEU and ASN (P = 0.59 and P = 0.59; Table S1), with the latter exhibiting the same non-monotonic relationship (Figure 3D) that we observed in the Perlegen data (Figure 1D). However, the differences between the populations are not significant, which we hypothesize is due to the smaller number of SNPs that we analyzed compared with the Perlegen data.

## Discussion

In this study, we have explored whether local recombination rate is associated with allele frequency differentiation across human populations when examined on a genome-wide scale. The negative correlation we find in the Perlegen data set (the larger of the two data sets we analyzed) corresponds to an average decrease of 4% in *F*_ST_ for every 1 cM/Mb increase in recombination rate. This correlation is mostly driven by the differentiation between African and non-African populations, where the decrease in *F*_ST_ is 5% for every cM/Mb. The differentiation of European and East Asian populations shows a qualitatively different, inverse U-shaped relationship with recombination rate. These results are present in both the data sets we analyzed, and unlike similar results for nucleotide diversity are not sensitive to the mutagenic effect of recombination. By considering only data sets that have been uniformly-ascertained, we also ruled out the possibility that the correlation is due to ascertainment biases correlating with recombination rate.

We considered various explanations for these observations, all involving natural selection. We first considered evolution favoring higher recombination rate in functionally important elements, which could potentially contribute to the higher recombination rate observed in genes, and which could also generate a correlation between recombination rate and allele frequency differentiation. However, we realized that this would generate a correlation in the opposite direction to what we observe: This is expected to result in a higher recombination rate in functionally important regions, which exhibit higher differentiation on average (Figure 2), leading to a positive correlation between the two, while we observe the opposite pattern.

The only force we could identify that can explain the observation of a negative correlation between recombination rate and *F*_ST_ is directional selection; that is, hitchhiking linked to positively selected alleles (sweeps) or background selection linked to negatively selected alleles. Evidence for this explanation comes from the stronger negative correlation between population differentiation and recombination rate in coding regions, though we did not have enough data to establish the difference between coding SNPs and the rest of the genome with high statistical significance. Hitchhiking of recent, geographically localized selective sweeps, after the split of African and non-African populations is a potential explanation of these results, especially considering the magnitude of the effect we observed, since it is expected to increase population differentiation and to have a more marked effect in regions of lower recombination. Alternatively, background selection also has the potential to increase population differentiation, because it is expected to decrease within-population diversity at regions linked to loci under negative selection, which will have a more marked effect in regions of lower recombination.

The different nature of the effect these two selective forces have on population differentiation should make it possible to distinguish them in finer-scale studies [41]. Specifically, selective sweeps are expected to act relatively quickly and to affect large distance scales, whereas background selection is expected to act slowly on slightly deleterious alleles, suggesting that these two forces will operate on different size scales. Thus, it may be possible to distinguish their impact by repeating our analyses at different distance scales once a genetic map with higher resolution becomes available.

A striking observation in our study is the qualitatively different relationships between recombination rate and allele frequency differentiation for different pairs of populations, suggesting that selection has acted in different ways over different epochs of history. A possible explanation for the stronger correlation observed between African and non-African populations, compared to that between Europeans and East Asians, is the smaller effective population size in non-African population history compared with African history since they diverged. Although in general a reduced effective population size makes selection less efficient, it can increase the impact of background selection on patterns of genetic variation since weakly deleterious mutations are less efficiently purged from the population, thereby reaching higher frequencies which results in more extensive background selection when they are purged [32],[53]. More background selection and less efficient positive selection in populations with smaller size may in principle explain the qualitatively different relationship that we observed between recombination rate and allele frequency differentiation of European and East Asian populations vs. African and non-African populations. While this explanation remains speculative, a recent study supports this possibility by showing that there are proportionally more deleterious mutations segregating in Europeans than in African Americans [54].

Another scenario that could potentially produce different *F*_ST_ patterns between different pairs of populations is if selective sweeps were shared to different extents across populations. When an allele that arose in one population and is under selection enters a second population via migration, *F*_ST_ at linked neutral sites can actually be *reduced* between the two populations since hitchhiking effectively takes variation from the between-population component and injects it into the within-population component [43],[55],[56]. Such global selective sweeps predict a *positive* correlation between *F*_ST_ and recombination rate. Europeans and East Asians exchanged genes more recently than both did with Africans, and hence a larger fraction of selective sweeps are expected to be shared between these two populations, introducing a component of positive correlation between *F*_ST_ and recombination rate. The signature of global selective sweeps is expected to decay differently with genetic distance from the selected site than the decay due to local sweeps [56], and thus in principle could result in an inverse U-shaped relationship between *F*_ST_ and recombination rate. A limitation of this explanation for our observations, however, is that the phenomenon of reduced *F*_ST_ due to a global selective sweep has only been demonstrated for populations that are much more diverged than human populations [56].

**Figure 4:**
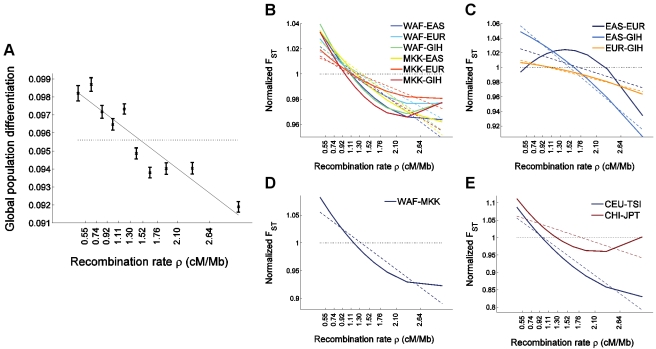
The relationship between population differentiation and recombination rate in the larger set of HapMap 3 populations. We placed 1,326,404 autosomal HapMap 3 SNPs (release 2) [57] into 10 bins according to recombination rate and estimated for each bin (A) global population differentiation between all 11 populations, and (B–E) population differentiation between pairs of populations. To avoid clutter, (B–E) depict only the linear (dashed lines) and quadratic (solid lines) regression of *F*_ST_ estimates as a function of the median recombination rate in each bin and partition the populations-pairs as follows: (B) *F*_ST_ between an African and a non-African population, where a negative correlation is observed with recombination rate, and where the quadratic regression is convex, (C) *F*_ST_ between a population of European, East Asian, or South Asian ancestry and a second population of a different one of these three ancestries, which shows a concave quadratic regression for all pairs of populations, and which recapitulates the result observed between North Europeans and East Asians in the uniformly-ascertained datasets (Figure 1D and Figure 3D). (A weaker phenomenon is observed for the South Asian GIH sample, which may be due to this population being somewhat related to both Europeans and East Asians [70], thereby confounding the North European–East Asian signal), (D) *F*_ST_ between two African populations, which shows a much steeper linear regression compared to intercontinental *F*_ST_, as well as a convex quadratic regression, and (E) *F*_ST_ between closely-related non-African populations (within either Europe or East Asia; genome-wide *F*_ST_<0.008), showing a very steep linear regression and a convex quadratic regression. *F*_ST_ based on all SNPs in all bins combined is presented as a horizontal dotted line and is equal to 1 in panels B–E since these present normalized *F*_ST_ values obtained by dividing each value by the genome-wide *F*_ST_ for the same pair of populations. Population codes are as follows: WAF (“West African”) is a combined sample of YRI (Yoruba in Ibadan, Nigeria) and LWK (Luhya in Webuye, Kenya); EAS (“East Asia”) is a combined sample of CHB (Han Chinese in Beijing, China), CHD (Chinese in Metropolitan Denver, CO, USA), and JPT (Japanese in Tokyo, Japan); EUR (“Europe”) is a combined sample of CEU (ancestry from Northern and Western Europe) and TSI (Toscani in Italia); GIH is a sample of Gujarati Indians in Houston, TX, USA; MKK is a sample of Maasai in Kinyawa, Kenya; and CHI (Chinese) is a combined sample of CHB and CHD.

To further study the pattern observed between different pairs of populations, we explored the relationship between *F*_ST_ and recombination rate in additional populations by studying data from HapMap 3, which genotyped 1,184 individuals from 11 populations [57]. A concern with the HapMap 3 data for our analysis is that the genotyping was carried out using SNP arrays (Affymetrix Human SNP array 6.0 and Illumina Human1M) that are affected by SNP ascertainment biases in a way that correlates with recombination rate [9]. Nevertheless, we observe the same qualitative results in the HapMap 3 data: We replicated our finding of a very strong negative correlation between *F*_ST_ and recombination rate, with an average decrease of 3% in global *F*_ST_ between all 11 populations for every 1 cM/Mb increase in recombination rate (Figure 4A). For pairwise *F*_ST_, the same observations we made with the Perlegen data are also observed, with a quadratic regression being concave only for inter-continental *F*_ST_ between European and Asian populations (Figure 4B and 4C).

In addition to qualitatively replicating our findings, analysis of HapMap 3 data allows us to generalize them to additional populations. A striking result is that the relationship between *F*_ST_ and recombination rate is stronger for *F*_ST_ between pairs of closely-related populations, whether within or outside Africa: *F*_ST_ between a West African sample and Maasai (of mixed West African and East African ancestry [57]) decreases by an average of 6% for every 1 cM/Mb (Figure 4D), *F*_ST_ between Italians and individuals of North-Western European ancestry decreases by 10% for every cM/Mb (Figure 4E), and *F*_ST_ between Japanese and individuals of Chinese ancestry decreases by 4% (Figure 4E). In view of the large effective population size in recent human history since each of these pairs of populations have split, these observations support the possibility that the different patterns observed between different pairs of populations are due to natural selection operating more efficiently in the context of larger population sizes. We observed a weak convex relationship with recombination rate for *F*_ST_ between closely-related populations in a quadratic regression analysis (Figure 4D–4E), which is intriguingly opposite to what was observed between Europeans and Asians (Figure 1D and Figure 4C). On the other hand, these observations do not seem to support the possibility that the different patterns are due to selective sweeps being shared to different extent across different pairs of populations since the level of gene flow between HapMap 3 closely-related populations likely have had been higher than that between continents. These results, while interesting, should be viewed with caution due to the confounder of ascertainment bias. It will be possible to test these observations further by analyzing data from the 1000 Genomes Project, where whole-genome sequencing will generate data that is largely free of ascertainment bias for many of the HapMap 3 populations as well as additional populations [58].

The approach presented in this study allows not only a comparison of the effect selection has on allele frequency differentiation at different historical times, but also a comparison across different compartments of the genome. Repeating the analysis on Perlegen “class A” X-linked SNPs (to contrast with the autosomal analyses we report above), we observed a very significant correlation between global population differentiation of X-linked SNPs and recombination rate, with a correlation coefficient of −0.86 (P = 0.001) when partitioning the data into 10 bins (Figure S2). The small number of X-linked SNPs available for analysis (26,074) does not allow any conclusive results in comparison with the autosomes. However, the relationship between X-linked population differentiation and recombination rate is suggestively more marked than the relationship we observed for autosomal population differentiation with linear regression predicting a decrease of 24% in *F*_ST_ for every 1 cM/Mb increase in recombination rate (compared with 4% predicted for the autosomes). If this suggestive result is verified, it will point to natural selection playing more of a role in allele frequency differentiation on chromosome X than on the autosomes. This observation is especially interesting in light of our recent finding that chromosome X exhibits higher allele frequency differentiation between Africans and non-Africans than would be expected from ¾ the effective population size of the autosomes [5]. If natural selection operated more powerfully in some sense on chromosome X than on the autosomes during the human dispersal out of Africa, it could be a plausible explanation for the increased frequency differentiation on chromosome X. From a theoretical point of view, we are not aware of a model that can explain how the effects of selection could be amplified on chromosome X relative to the autosomes during the out of Africa dispersal. However, it should be possible to test this hypothesis empirically by analyzing data from whole-genome sequencing, which will allow a much more accurate analysis for chromosome X.

More generally, these results show that comparing the relationship of differentiation and recombination rate between different genomic regions and in different populations is a promising direction to be explored in future studies with larger data sizes. In addition to using this approach to study natural selection, by extrapolating the prediction of population differentiation to “infinite” recombination rate it might be possible to predict the level population differentiation that is due to genetic drift alone, separate from the effect of selection, since every nucleotide becomes independent of selection at nearby sites. (Population differentiation is still affected by natural selection at the sites directly under selection.) Models of demographic history would be expected to be more accurate if one used the prediction of *F*_ST_ for high recombination rate, rather than the genome-wide average. This is in the same spirit of studies targeting regions of high recombination rate and far from functional elements to infer human demographic history [10], [59]–[61]. To illustrate this type of analysis, we note that for global *F*_ST_ in the Perlegen data set, the genome-wide estimate is 0.121, while it is 0.113 for the SNPs in the bin with 10% highest recombination rate (Figure 1). For studies that attempt to infer history—for example, the time of split between human populations—this adjustment in *F*_ST_ would have a profound effect. The effect would be even larger if extrapolating to higher recombination rates.

A limitation of this study is the genetic map available. We chose to use a pedigree-derived human genetic map [48] since it is based on the direct observation of recombination events. Population-based genetic maps that are based on patterns of LD in a population [62]–[65] are sensitive to the increase in LD due to natural selection [66],[67], the very force which effect we sought to analyze. In future studies of these phenomena, it will be valuable to use genetic maps that are based on the direct observation of recombination events and that have a higher spatial resolution. As recombination rates vary on a fine-scale [68] and background selection may occur at a somewhat different genetic distance scale than hitchhiking, a finer-scale map should allow a better characterization of the relationship between population differentiation and recombination rate and improved statistical power in capturing its causes. Supporting this view, a recent study examining the correlation between *nucleotide diversity* and recombination rate in *Drosophila* found it to be non-significant when recombination rate was estimated in 2 Mb windows and to be significant when finer-scale heterogeneity in recombination rate was considered in the analysis [17].

In conclusion, we have shown that genome-wide human population differentiation in allele frequencies is significantly correlated with recombination rate on a megabase scale, demonstrating that natural selection has had a profound effect on allele frequency distributions averaged over the last hundred thousand years. While these results likely reflect the effects of hitchhiking and background selection, disentangling the strengths of these two forces will require extending the analyses presented in this paper. One important direction is to use genetic maps that have fine spatial resolution, which may shed light on the detailed distribution of selective coefficients that have shaped allele frequency differentiation. A second direction in which these results can be extended is to compare more populations of continentally diverse ancestry. This should facilitate an exploration of the relationship between recombination rate and population differentiation during different epochs of human evolution, and should allow a better understanding of how demographic history has shaped the impact of natural selection on patterns of human genetic variation.

## Methods

### Perlegen data

To examine the correlation between SNP allele frequency differentiation and recombination rate in a way that is not sensitive to the confounder of recombination rate-dependent SNP ascertainment, we limited our main analysis to autosomal Perlegen “class A” SNPs [46]. These SNPs, encompassing ∼69% of SNPs in the Perlegen data set [46], were uniformly discovered by array-based resequencing of 24 human samples of diverse ancestry from the NIH Polymorphism Discovery Resource [47]. All SNPs discovered in this way were genotyped in 24 European Americans, 23 African Americans, and 24 Han Chinese from the Los Angeles area, which were all unrelated to each other and also unrelated to the individuals in the discovery set [46]. We used liftOver (http://genome.ucsc.edu/cgi-bin/hgLiftOver) to convert genomic positions from build 34 (hg16) used in the Perlegen data set to build 35 (hg17) and discarded SNPs for which this conversion failed. We then estimated recombination rate around each SNP and discarded SNPs for which it could not be accurately estimated (see below). Following these filters, we were left with 1,110,338 autosomal SNPs with recombination rate estimates. Out of these, we also identified 21,391 coding SNPs according to the UCSC Known Genes track [69] for separate analysis.

### Uniformly ascertained HapMap data

We replicated our results in a data set of SNPs that were uniformly ascertained as polymorphic in exactly two chromosomes of the same ancestry and genotyped in all HapMap samples [5], including 60 West Africans from Ibadan, Nigeria (YRI), 60 European Americans from Utah, USA (of North European ancestry; CEU), and 90 East Asians (ASN; 45 Han Chinese from Beijing, China (CHB) and 45 Japanese from Tokyo, Japan (JPT), which we pooled for analysis). We considered only samples that were unrelated to each other and also unrelated to the few individuals in the discovery sets. In a previous study using these uniformly ascertained SNP sets, we were interested in learning about features of human genetic variation that are not due to known effects of natural selection, and hence in that study we filtered genes, conserved non-coding loci, and regions that were putatively affected by selective sweeps [5]. As we were interested in exploring the effect of selection on population differentiation in the current study, we relaxed these filters. Furthermore, we pooled together SNPs discovered using different pairs of chromosomes since we were only concerned with the ascertainment being uniform genome-wide and independent of LD, which resulted in 248,886 SNPs for which we could accurately estimate recombination rate.

### Recombination rate estimation

We determined recombination rate around each SNP based on the deCODE genetic map [48]. We considered a three megabase (Mb) window centered on each SNP and determined the genetic length in centimorgan (cM) for each window by subtracting the genetic position of the start from that of the end position of the window. The genetic position of the start and end positions were each determined by linearly interpolating, according to distance, the genetic position of the nearest marker in the genetic map on each side. We discarded the SNP from further analysis in cases where (i) the window's start position is before the chromosome start position, (ii) the window overlaps a centromere, or (iii) the window overlaps a telomere. To place SNPs into bins according to recombination rate, we chose bin boundaries such that an equal number of SNPs fell into each bin. We also repeated the main analyses while modifying the window length around each SNP from 3 Mb to either 1 Mb or 5 Mb and obtained similar results (Figure S3 and Figure S4). Similar results were also obtained when we masked out an additional 5 Mb of each telomeric region and on each side of each centromere, showing that our results are not explained by unusual patterns at these regions (Figure S5).

### Allele frequency differentiation estimates

To estimate pairwise allele frequency differentiation between populations, we used the *F*_ST_ statistic as formulated in ref. [4]. In the context of the current study, these *F*_ST_ estimates are almost identical to the estimates obtained based on the estimator of Weir and Cockerham [1]. Global allele frequency differentiation was calculated as the average *F*_ST_ over all population-pairs. We estimated pairwise and global *F*_ST_ across all SNPs in each recombination rate bin. We also estimated *F*_ST_ standard errors (for presentation in Figure 1, Figure 2, Figure 3, Figure 4, and Figures S1, S2, S3, S4, S5) based on bootstrapping 1,000 random sets of SNPs from each bin using the moving block bootstrap (MBB) [49]–[51], randomly resampling contiguous runs of SNPs to take into account the effect of correlation between SNPs in LD [4]. We applied the same bootstrapping procedure to estimate *F*_ST_ for all SNPs irrespective of bins, and used the standard errors for z-tests to determine whether the genome-wide *F*_ST_ values are different, e.g. between cSNPs and ncSNPs.

### Bootstrapping framework

We randomly resampled 1,000 data sets of SNPs from the set of all SNPs using MBB. For each of these sets, we started by stratifying resampled SNPs into bins according to their recombination rate, and then repeated the procedure that was used to analyze the original data set. Specifically, we estimated *F*_ST_, and then estimated the correlation and regression between *F*_ST_ in a bin and the bin's median recombination rate *ρ*. Averaging across all 1,000 resamplings, we obtained accurate estimates for several correlation and regression statistics: (i) The coefficients of the linear regression *F*_ST_ = *b_0_*+*b_1_ρ*, (ii) the correlation coefficient across bins, (iii) a t-statistic for the significance of the linear coefficient of the above regression, and (iv) a normalized regression coefficient *b_1_/b_0_*. We estimated standard errors of these statistics as the standard deviation across resamplings. Hence, these standard errors account for the problem that nearby SNPs do not provide independent observations due to LD.

Based on averaged estimates across resamplings and their standard errors, we performed two-sided z-tests for the significance of each statistic (Table 1 and Table S1). We also compared estimates from analyses of different data: Testing for a significantly higher ratio of the regression coefficients (*b_1_*/*b_0_*) for cSNPs than for ncSNPs (one-sided z-test) and for a significantly different ratio between different pairs of populations (two-sided z-test). We also reported hypothesis testing for whether *b_0_* and *b_1_* by themselves are higher in cSNPs than ncSNPs (one-sided z-test).

## Supporting Information

Figure S1 Relationship between population differentiation and recombination rate for different minor allele frequencies (MAFs). We divided 1,110,338 SNPs into 4 categories according to their MAF: (A) MAF≤0.125, (B) 0.125<MAF≤0.25, (C) 0.25<MAF≤0.375, and (D) 0.375<MAF (≤0.5). For each category, we partitioned SNPs into 10 bins according to the recombination rate around each SNP and presentation is similar to Figure 1A. Linear regression of *F*_ST_ estimates as a function of the median recombination rate in each bin (solid line) is (A) 0.0855–0.0010ρ (B) 0.1222–0.0048ρ (C) 0.1371–0.0059ρ, and (D) 0.1434–0.0065ρ. The corresponding correlation coefficient estimates between *F*_ST_ and median recombination rate is (A) r = −0.691 (P = 0.0269), (B) −0.960 (P = 1.0×10^−5^), (C) −0.954 (P = 1.9×10^−5^), and (D) −0.865 (P = 0.0012). We emphasize that natural selection being the force behind the correlation between population differentiation and recombination rate can entail a (non-causal) relationship between MAF and recombination rates since selection changes allele frequencies. Nevertheless, the correlation of population differentiation and recombination rate is very significant for all categories of common SNPs (B–D). The results are not as significant, though a correlation is observed, for SNPs of low MAF (A), likely due to the effect of negative selection on allele frequencies.
(1.15 MB EPS)

Figure S2 Population differentiation in allele frequencies is inversely correlated with recombination rate on chromosome X. We placed 26,074 Perlegen “class A” X-linked SNPs into 10 bins according to the recombination rate in a 3 Mb window centered on each SNP. The x-axis of all panels indicates the recombination rate, with the values indicated on the ticks corresponding to the edges between the 10 bins. For each bin, at an x-axis position corresponding to the median recombination rate across the SNPs at that bin, the figure presents global population differentiation between African Americans, Europeans, and Chinese. Error bars indicate ±1 standard error, which is estimated based on 1,000 moving block bootstraps over the SNPs in the bin. Linear regression of *F*_ST_ estimates as a function of the median recombination rate in each bin is also presented (solid line), corresponding to 0.2272–0.0552ρ. The corresponding correlation coefficient between *F*_ST_ and median recombination is r = −0.860 (P = 0.0014). For comparison, population differentiation based on all SNPs in all bins combined is also presented (horizontal dotted line).
(0.01 MB EPS)

Figure S3 Results when recombination rate is estimated in a 5 Mb window. The figure mirrors Figure 1, except for the use of a 5 Mb window centered on each SNP for estimating its recombination rate (Methods).
(1.12 MB EPS)

Figure S4 Results when recombination rate is estimated in a 1 Mb window. The figure mirrors Figure 1, except for the use of a 1 Mb window centered on each SNP for estimating its recombination rate (Methods).
(1.15 MB EPS)

Figure S5 Results when filtering regions near centromeres and telomeres. In the main analyses a SNP was discarded if the 3 Mb around it overlaps either a centromere or a telomere. To more cautiously account for the possibility of our results being sensitive to centromeric or telomeric regions, we repeated the analysis while also discarding SNPs for which the 3 Mb window around them is within 5 Mb of such a region (namely, the SNP is within 6.5 Mb of such a region). The figure mirrors Figure 1, except for the application of this additional filter, following which the SNPs were re-partitioned into 10 bins by recombination rate.
(1.15 MB EPS)

Table S1 Bootstrapped correlation and regression coefficient estimates of *F*_ST_ as a function of recombination rate in uniformly-ascertained subsets of HapMap. The table mirrors Table 1, except that we applied the analysis to a different data set of uniformly-ascertained subsets of HapMap SNPs. We analyzed global *F*_ST_ between all three HapMap populations, as well as pairwise *F*_ST_ between each pair of populations.
(0.07 MB DOC)
