# Supplementary material for: Human Population Differentiation Is Strongly Correlated with Local Recombination Rate
Source: PLoS Genet. 2010 Mar 26;6(3):e1000886. doi: 10.1371/journal.pgen.1000886 (PMC2845648; doi:10.1371/journal.pgen.1000886)
Supplement: Table S1 — Bootstrapped correlation and regression coefficient estimates of F ST as a function of recombination rate in uniformly-ascertained subsets of HapMap. The table mirrors Table 1, except that we applied the analysis to a different data set of uniformly-ascertained subsets of HapMap SNPs. We analyzed global F ST between all three HapMap populations, as well as pairwise F ST between each pair of populations. (0.07 MB DOC) [file pgen.1000886.s006.doc]

|  | **Global *F*_ST_** | **Pairwise *F*_ST_** | | |
| --- | --- | --- | --- | --- |
|  |  | **YRI vs. CEU** | **YRI vs. ASN** | **CEU vs. ASN** |
| Number of SNPs | 248,886 | 248,886 | 248,886 | 248,886 |
| *b_0_* ± stderr (p-value) | 0.1472±0.0016  (<<10^-12^) | 0.1538±0.0019  (<<10^-12^) | 0.1819±0.0021  (<<10^-12^) | 0.1063±0.0020  (<<10^-12^) |
| *b_1_* ± stderr (p-value) | -0.0026±0.0009  (0.0039) | -0.0027±0.0012  (0.0244) | -0.0047±0.0013  (3.0x10^-4^) | -0.0006±0.0011  (0.5854) |
| *r* ± stderr (p-value) | -0.4557±0.1415  (0.0013) | -0.4093±0.1554  (0.0084) | -0.5655±0.1283  (1.0x10^-5^) | -0.0680±0.1244 (0.5846) |
| *t* ± stderr (p-value) | -1.5239±0.6146  (0.0132) | -1.3350±0.5935  (0.0245) | -2.0432±0.6733  (0.0024) | -0.1968±0.3610  (0.5856) |
| *b_1_*/*b_0_* ± stderr | -0.0173±0.0059 | -0.0177±0.0074 | -0.0258±0.0069 | -0.0055±0.0103 |

#### 
